# OCT-Derived Plaque Morphology and FFR-Determined Hemodynamic Relevance in Intermediate Coronary Stenoses

**DOI:** 10.3390/jcm10112379

**Published:** 2021-05-28

**Authors:** Mariusz Tomaniak, Dorota Ochijewicz, Łukasz Kołtowski, Adam Rdzanek, Arkadiusz Pietrasik, Jacek Jąkała, Magdalena Slezak, Krzysztof P. Malinowski, Martyna Zaleska, Jakub Maksym, Piotr Barus, Tomasz Roleder, Krzysztof J. Filipiak, Grzegorz Opolski, Janusz Kochman

**Affiliations:** 1 First Department of Cardiology, Medical University of Warsaw, 02-097 Warsaw, Poland; dorota.ochijewicz@gmail.com (D.O.); lukasz@koltowski.com (Ł.K.); ardzanek@poczta.wp.pl (A.R.); apietrasik@tlen.pl (A.P.); zaleskamartyna@gmail.com (M.Z.); jakub.maksym@gmail.com (J.M.); piotrbarus@op.pl (P.B.); krzysztof.filipiak@wum.edu.pl (K.J.F.); grzegorz.opolski@wum.edu.pl (G.O.); jkochman@wum.edu.pl (J.K.); 2 Krakow Cardiovascular Research Institute, 30-055 Krakow, Poland; jacek.jakala@kcri.org (J.J.); mslezak@kcri.org (M.S.); krzysztof.malinowski@doctoral.uj.edu.pl (K.P.M.); tomaszroleder@gmail.com (T.R.); 3 Regional Specialist Hospital, Research and Development Center, 51-124 Wroclaw, Poland

**Keywords:** optical coherence tomography, fractional flow reserve, stable coronary artery disease

## Abstract

Background: optical coherence tomography (OCT) might allow identifying lesion features reportedly associated with plaque vulnerability and increased risk of clinical events. Previous studies on correlation between OCT and functional lesion significance indices reported contradictory results, yet integration of complementary information from both modalities is gaining increased interest. The aim of the study was to compare plaque morphology using OCT in hemodynamically relevant vs. non-relevant lesions by fractional flow reserve (FFR). Methods: consecutive patients with intermediate grade coronary stenoses by angiography were evaluated by both FFR and OCT in this single-center study. Stenoses were labeled hemodynamically relevant in case of the FFR ≤ 0.80. Minimal lumen area (MLA), fibrous cap thickness (FCT), minimal cap thickness over the calcium, angle of the calcium, and necrotic core within the lesions were evaluated. Results: a total of 105 patients (124 vessels) were analyzed. Of them, 65 patients were identified with at least one lesion identified as hemodynamically relevant by FFR (72 vessels, 58.1%). Lesions with FFR ≤0.80 presented with lower mean and minimal lumen area (3.46 ± 1.29 vs. 4.65 ± 2.19, *p* =0.001 and 1.84 ± 0.97 vs. 2.66 ± 1.40, *p* = 0.001) compared to patients with FFR > 0.80. No differences were found between groups in the mean and minimal FCT, mean, and maximal necrotic core, calcium angle, as well as the overall rate of calcified and lipid plaques. Conclusion: hemodynamic relevance of intermediate grade lesions correlated moderately with the luminal assessment by OCT. No differences were identified in the plaque morphology between relevant and non-relevant coronary stenoses by FFR.

## 1. Introduction

Fractional flow reserve (FFR) represents a guideline-recommended modality for functional assessment of intermediate grade coronary stenosis [1,2]. The ability of FFR to predict not only ischemia-related symptoms, but also risk for acute coronary syndromes (ACS), often related to plaque rupture and/or erosion with subsequent coronary thrombosis, represents a subject of ongoing research [3,4,5]. Atherosclerotic plaques with large necrotic cores have been associated, with higher risk of rupture and cause ACS [6]. Emerging evidence also suggests a relationship between plaque volume, atherosclerotic plaque characteristic, and the extent of ischemia, as assessed by FFR [4,5,7].

However, there is a substantial heterogeneity in the results of studies addressing correlation between FFR- and optical coherence tomography (OCT)-based quantitative and qualitative measurements [3,8,9,10]. The relationship between FFR and OCT-detected plaque components, including features suggestive of plaque vulnerability and subsequent adverse clinical events, such as thin cap fibroatheroma (TCFA), remains not sufficiently understood [11,12,13].

Concurrently, integration of data acquired from both modalities is gaining an increasing interest, as exemplified by recently validated FFR-derived from OCT pullback, namely OCT-based FFR (OFR) [14].

Given this background, we aimed to explore the association between FFR measurements and OCT-derived plaque characteristics by comparing plaque morphology using OCT in hemodynamically relevant vs. non-relevant lesions by FFR.

## 2. Materials and Methods

### 2.1. Study Population

In this single-center, non-randomized, longitudinal study, patients with stable coronary artery disease (CAD) and intermediate grade coronary were evaluated with both FFR and OCT. Stenoses were labeled hemodynamically relevant in case of the FFR ≤ 0.80.

The inclusion criteria involved: presentation with stable CAD: prevalence and severity of chest pain symptoms ranked according to the Canadian Cardiovascular Society classification (CCS 2-3) or positive ischemia test (exercise test or single photon emission tomography (SPECT)), age >18 years, intermediate grade coronary stenoses defined as stenosis of 40–80% [15], as assessed by visual estimation in angiography, both FFR and OCT examination performed in the same lesion. Exclusion criteria comprised: left main disease, ostial right coronary lesion, bypass graft lesions, contraindications to adenosine administration, hemodynamic instability, acute or chronic renal insufficiency (serum creatinine level >1.5 mmol/L), and pregnancy.

### 2.2. Optical Coherence Tomography Imaging

OCT images were obtained with a commercially available frequency domain OCT imaging system (Abbott, C7XR Dragonfly TM, LightLab Imaging Inc., MA, USA), using the non-occlusive flushing technique. OCT pullbacks were analyzed at 0.2 mm intervals by the independent core laboratory Krakow Cardiovascular Research Institute (KCRI), Krakow, Poland, using the proprietary LightLab off-line analytical software by two analysts blinded to the angiographic data and patients’ clinical characteristics.

### 2.3. Fractional Flow Reserve Examination

Coronary pressure was measured using a 0.014-inch pressure guide wire (St. Jude Medical, Minneapolis, MN, USA). Maximal hyperemia was induced by intravenous adenosine infusion administered at 140 µg/kg/min through a large peripheral vein. FFR was calculated using the following formula: mean hyperemic distal coronary pressure/mean aortic pressure. The stenosis was considered functionally significant when the FFR was ≤0.80.

### 2.4. OCT Definitions

**Figure 1:**
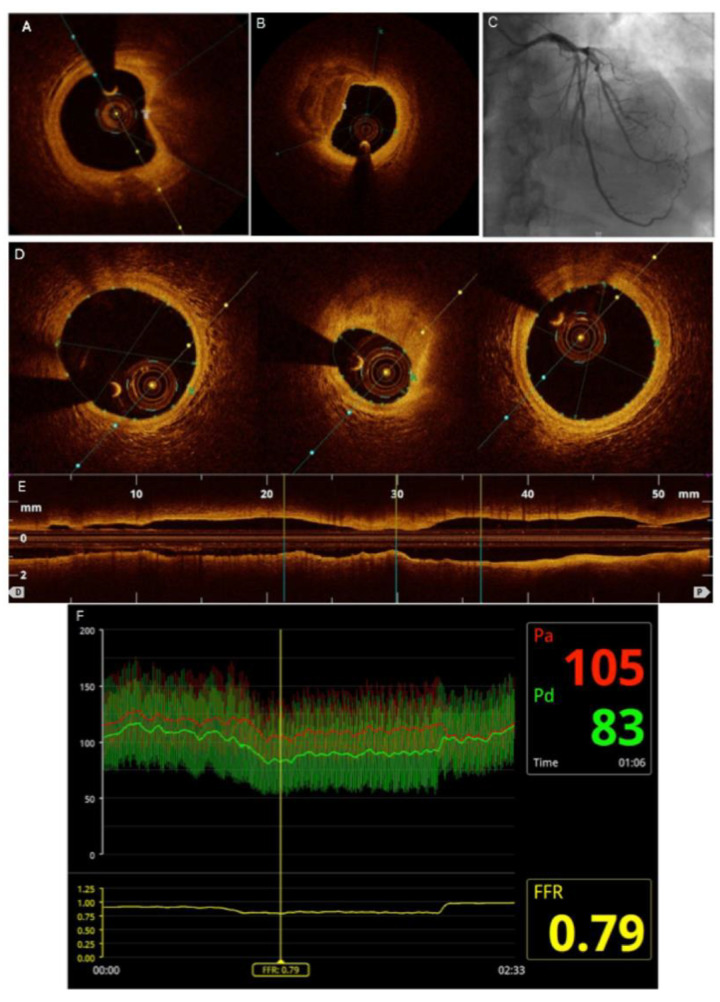
(**A**) Optical coherence tomography measurement of the minimal fibrous cap thickness (11 µm) and the necrotic core angle (69.1°) in the thin cap fibroatheroma (TCFA). (**B**) Fibrocalcific plaque with fibrous cap thickness of 11µm and the angle of 126°). (**C**) Angiographic view of an intermediate grade stenosis of the left anterior descending artery (fractional flow reserve measurement 0.79). (**D**) Optical coherence tomography cross sectional images with measured lumen dimensions at various locations: distal reference segment, minimum lumen area, proximal reference segment. (**E**) Longitudinal OCT reconstruction of the artery showing the stenosis and locations of OCT cross sectional images. (**F**) Fractional flow reserve (FFR) measurement of the corresponding coronary stenosis; Pa represents the pressure proximal to the lesion while Pd indicates the pressure distal to the lesion; FFR value is calculated as the ratio of Pd and Pa.

OCT images analyses were performed in compliance with the recently published consensuses [16,17,18], by analysts blinded to patient clinical, angiographic characteristics and the FFR results. The site of the minimal lumen area (MLA) was defined as the segment with the smallest lumen area. Measurements of reference lumen area were performed at the largest lumen proximal or distal to a stenosis but within the same segment (usually within 10 mm of the stenosis, with no major intervening branches). Plaque morphology was analyzed at the site of MLA in at least three consecutive frames and was classified into fibrous, calcified or lipid-rich. Fibrous plaque had high backscattering and a relatively homogeneous OCT signal. Calcified plaque contained fibrous tissue with calcium that appeared as a signal-poor or heterogeneous region with a sharply delineated border and the calcium angle (the circumference of the calcium covering the lumen and presented in degrees) was measured. The lipid-rich plaque was defined as the signal-poor region with poorly delineated borders covered by a fibrous cap. A plaque was considered lipid-rich if lipid was present for more than 90° in any cross-sections of the plaque [19,20]. The lipid angle (the circumference of the lipid-rich plaque and presented in degrees) was measured. Fibrous cap thickness (FCT) was the distance between the arterial lumen and the inner border of the lipid or calcium pool. The FCT of each lipid-rich plaque was measured first at 0.2-mm intervals over the lipid plaque and then 3 times at its thinnest part at each cross-section, and the average value was calculated [20] Thin-cap fibroatheroma (TCFA) was defined as a lipid-rich plaque with minimal FCT < 65 μm. The examples of both FFR and OCT assessments are presented in Figure 1.

### 2.5. Quantitative Coronary Angiography

Two orthogonal views of every major coronary vessel were recorded. Off-line quantitative coronary angiography (QCA) analysis was performed using the Cardiovascular Angiography Analysis System 5.11.1 (Pie Medical Imaging Systems, Maastricht, Netherlands), by an independent core laboratory (KCRI, Krakow, Poland). Analyses were performed by experienced readers, blinded to the patient, FFR and OCT data.

### 2.6. Ethics

The study was approved by the local research ethics committee and was conducted in accordance with Declaration of Helsinki. All patients provided a written informed consent.

### 2.7. Statistical Analysis

The Shapiro–Wilk test was used to analyze the continuous data distribution. Normally distributed values were presented as a mean ± standard deviation. Non-normally distributed values were presented as median with 25th and 75th percentile (IQR—interquartile range). One-way ANOVA was used to compare normally distributed data, and the Mann-Whitney test was used to compare non-normally distributed data. The chi square test (or Fishers’ exact test) and was used for comparison of categorical data. FFR and OCT measurements was assessed by Pearson correlation coefficient. Clinical outcomes were analyzed using the Kaplan–Meier method and compared using the log rank test. All statistical tests were two-sided and the *p* value of 0.05 was considered statistically significant. Statistical analyses were performed using SPSS 25.0 IBM software (IBM, Armonk, NY, USA).

## 3. Results

A total of 105 patients (124 lesions) were analyzed. Overall, 81.9% (86) of enrolled patients were male, 32.4% (34) were diabetic, 83.8% (88) were hypertensive, and 10.5 % (11) had a history of chronic kidney disease (Table 1).

There were 65 patients with at least one coronary artery lesion identified as hemodynamically relevant by FFR. Patients with at least one lesion with FFR ≤ 0.8 were more frequently hypertensive (Table 1).

Bifurcations were found in 41.7% and 40.4% of lesions with FFR values ≤ 0.8 and >0.8, respectively.

Lesions with FFR values ≤ 0.8 were more frequently located in left anterior descending artery (LAD); were longer (28.4 ± 13 vs. 17.29 ± 7.5, *p* = 0.001), with greater diameter stenosis (53.3 ± 8.7 vs. 47.6 ± 8.6, *p* = 0.036), and smaller reference vessel diameter: 2.75 ± 0.43 vs. 3.18 ± 0.77, *p* = 0.001), compared with lesions with FFR > 0.8.

No significant differences were found between patients with lesions with FFR values ≤ 0.8 and >0.8 in terms of remaining clinical and angiographic characteristics (Table 1 and Table 2).

**Figure 2:**
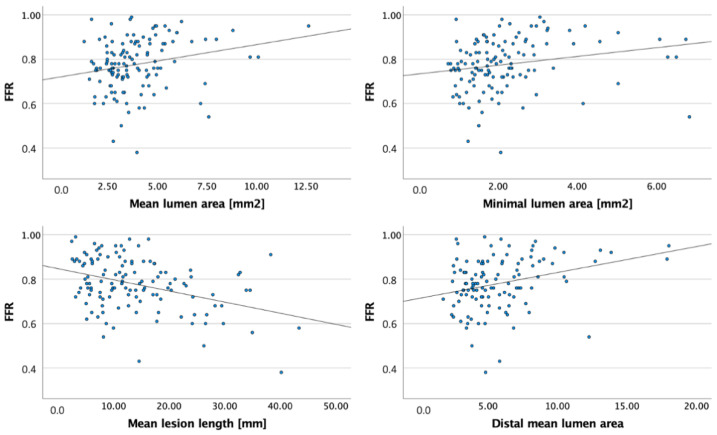
Scatter plots of fractional flow reserve and optical coherence tomography measurements. (FFR—fractional flow reserve).

By OCT, lesions with FFR ≤ 0.80 presented with smaller mean and minimum lumen area (3.46 ± 1.29 vs. 4.65 ± 2.19, *p* < 0.001 and 1.84 ± 0.97 vs. 2.66 ± 1.4, *p* < 0.001), smaller proximal (7.27 ± 2.73 vs. 9.73 ± 5.31, *p* = 0.002) and distal reference lumen area (4.89 ± 1.93 vs. 6.85 ± 3.63, *p* < 0.001), had a greater mean lesion length (15.62 ± 9.42 vs. 11.8 ± 7.79, *p* = 0.018), compared to patients with FFR > 0.80 (Table 2). FFR measurements had moderate correlation with lesion length and weak correlation with mean and minimum lumen area and reference areas (Table 3, Figure 2).

There were no significant differences found between groups in the mean (0.11 ± 0.07 mm vs. 0.11 ± 0.07 mm, *p* = 0.882) and minimal FCT (0.10 ± 0.07 mm vs. 0.10 ± 0.08 mm, *p* = 0.905), mean and maximal lipid and calcium angle as well as minimal cap thickness over the calcium (Table 2). Although mean angle of the calcium had a trend towards higher values in the FFR ≤ 0.80 group (Table 2). 

Overall, at the site of MLA, similar rates of calcified (36.1% vs. 38.5%, *p* = 0.961) and lipid plaques (37.5% vs. 28.8%, *p* = 0.315) were found in hemodynamically relevant and non-relevant stenoses (Table 2).

**Figure 3:**
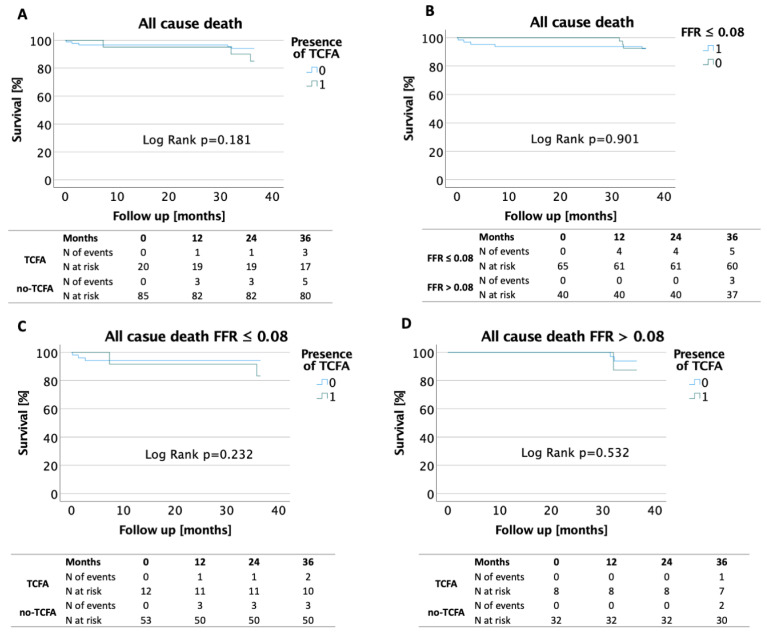
Kaplan–Meier survival curves (all cause death) A—in patients with and without presence of TCFA; B—in patients with FFR ≤ 0.80 and FFR > 0.80; C—in patients with FFR ≤ 0.80 with and without presence of TCFA; D—in patients with FFR > 0.80 with and without presence of TCFA (FFR–fractional flow reserve; TCFA–thin cap fibroatheroma).

There were no significant differences in the mortality rates between patients with or without the presence of TCFA irrespective of FFR values. The survival rates between patients with FFR ≤ 0.80 treated with PCI and patients with FFR > 0.8 was comparable (Figure 3).

## 4. Discussion

The main findings of the present investigation are summarized as follows:

1. OCT-derived MLA and mean lesion length demonstrated a weak to moderate correlation with hemodynamic relevance.
2. No significant differences were identified in the morphometric characteristics of coronary plaques in relation to FFR.

Our findings are consistent with some previous OCT studies in which minimum lumen area or percent area stenosis was associated with FFR, but not the plaque composition, such as the presence of thin cap fibroatheroma or the lipid angle [3,8,9,10,21,22,23]. Similar observations were also reported in studies employing iFR or three-dimensional quantitative coronary angiography-derived FFR indices, such as quantitative flow ratio (qFR), which confirmed a significant association between the minimum lumen area and iFR or qFR, respectively [24,25,26].

Although morphological characteristics of plaques, such as lipid arc and lipid length assessed by OCT presented a significant correlation with FFR in studies by Usui et al. and Lee et al. [21,27], no independent association with FFR in the model including OCT-derived MLA could be demonstrated. The present study also builds up on the recently published results from a smaller study by Burzotta et al. who, amongst 45 patients with CAD, found that a combination of different OCT parameters, such as MLA, % area stenosis, presence of thrombus or plaque ulceration could aid in prediction of significant FFR results [4].

The clinical importance of plaque morphology was first described in the PROSPECT trial [28]. Plaque burden and MLA, as detected by 3-vessel radiofrequency intravascular ultrasound imaging, were associated with an increased risk of developing future events. TCFA was shown to be an independent predictor of future non-culprit lesion related adverse cardiac events in patients with diabetes mellitus [29]. Morphological assessment of ruptured plaques revealed that fibrous cap thickness and a combination of large plaque burden and small lumen area result in ACS [30]. Although PCI for vulnerable coronary atherosclerotic plaques seems to be a safe procedure, a systemic pharmacotherapy rather than individual ‘plaque sealing’ remains the key intervention in the treatment of TCFA in non-flow limiting coronary lesions [31,32,33].

Furthermore, Reith et al. specifically in diabetic patients showed that FFR may predict potentially unstable lesions with minimal FCT ≤ 80 mm and could be associated with vulnerable, lipid-rich plaques morphology within lesion of intermediate severity by angiography [34]. Mechanistically oriented interpretation may suggest that mechanisms linking plaque characteristics with coronary flow regulation—possibly including the presence of endothelial dysfunction, inflammatory response, and transient microvascular dysfunction accompanying ACS—could hypothetically be visualized only in arteries of high cardiovascular risk patients, such as diabetics, while no clear differences could be observed in the general stable CAD population enrolled in the present investigation, in which the diabetic patients comprised 32% of patients. Given the small sample size, the results of our analysis should be interpreted with caution, mainly as confirming previous data on the correlation between anatomic features and hemodynamic impact of CAD. While no significant association was found between any of plaque components and FFR, there was a numerically higher calcification load in lesions with FFR < 0.8 as expressed by mean calcification angle. Furthermore, presented findings should be interpreted in light of different inclusion criteria among the studies on the topic [3]. In contrast to some prior studies addressing OCT and FFR correlations, our study did not exclude patients with ostial lesions, bifurcations, and tortuous vessels [3]. Importantly, based on the presented study, neither recommendation could be made supporting the use of imaging for evaluation of functional lesion severity.

FFR reportedly discerns not only lesions producing ischemia-related symptoms, but also identify lesions at high risk for future ACS or with a low risk for plaque rupture and coronary thrombosis that may be treated effectively with optimal medical treatment alone [35,36]. Among lesions with 40% to 80% luminal narrowing by visual estimation, up to half had FFR ≤ 0.8, corroborating the previous observations implying that factors beyond luminal stenosis might contribute to inducible ischemia [37]. The benefit of FFR-guided therapy has been hypothesized to be related to the association of local vasodilator reserve and features of plaque vulnerability. However, adverse cardiovascular events still occur in patients with functionally insignificant stenosis with the prevalence of approximately 9% at 2 years [2]. Since the identification of vulnerable plaques in these patients may stratify an additional risk, the association between hemodynamic relevance and plaque characteristics should be better explained. In our study the presence of TCFA did not affected survival rates in patients with FFR < 0.8. Nevertheless, it has to be noted that no intravascular imaging follow-up was performed and, thus, no firm conclusions can be drawn on the association between the plaque morphology and long-term survival in this study.

The imaging studies employing IVUS suggested that the volume of lipid plaque was significantly associated with the FFR value [38,39]. Nevertheless, the ability of IVUS to generate imaging of the entire thickness of the coronary artery wall permits a comprehensive evaluation of the plaque burden in contrast to OCT while imaging depth of OCT is still limited to 0.5–2.0 mm [16]. In the present study, the angle of necrotic core, calcium plaque, fibrous cap thickness, or cap thickness over the calcium did not correlate with FFR.

### 4.1. Future Directions

Our observations add to the currently understood need for multimodality, and/or hybrid approaches for coronary lesions evaluation. With the granularity of information provided by near-histology precise OCT imaging, development of further software that would integrate OCT imaging with functional lesion assessment indices for diagnostics, pre-procedural planning, and post PCI assessment, is anticipated [14]. Further studies with larger populations, aimed to examine the interplay between the plaque characteristics and FFR could facilitate development of such diagnostic tools.

### 4.2. Study Limitations

Several limitations of this study have to be considered. This is a single-center study with a relatively small sample size. The study is restricted only to stable CAD patients and these data cannot be extrapolated to patients with ACS due to different interpretation and treatment of coronary lesion morphology. OCT and FFR measurement accuracy might have been limited in some circumstances, such as ostial lesions, bifurcations, and tortuous vessels. The clinical results included all-cause mortality only, and further studies, including major cardiac adverse events (cardiovascular death, myocardial infarction, revascularization), as well as imaging and functional assessment of the lesion, are warranted.

## 5. Conclusions

Hemodynamic relevance of intermediate grade lesions correlated moderately with the luminal assessment by OCT. No differences were identified in the plaque morphology between relevant and non-relevant coronary stenoses by FFR.

## Figures and Tables

**Table 1:** Baseline characteristics in patients categorized according to presence of at least one lesion with FFR ≤ 0.8.

Baseline Characteristics			
	FFR ≤ 0.80 *	FFR > 0.80	*p*
	*n* = 65	*n* = 40	
Age (years)	63.83 ± 9.2	66.47 ± 10.1	0.172
Male	55 (84.62)	31 (77.5)	0.358
CCS 3	12 (18.46)	5 (12.5)	0.421
Diabetes mellitus	21 (32.31)	13 (32.5)	0.984
Hypertension	50 (76.92)	38 (95.0)	0.015
Dyslipidemia	42 (64.62)	27 (67.5)	0.762
Chronic kidney disease	8 (12.31)	3(7.5)	0.435
Chronic heart failure	12 (18.46)	4 (10.0)	0.241
Previous PCI	51 (78.46)	26 (65.0)	0.13
Previous CABG	4 (6.15)	1 (2.5)	0.625
Previous MI	37 (56.92)	20 (50.0)	0.489
TIA/stroke	2 (3.08)	1 (2.5)	0.853
Current smoking	14 (21.54)	4 (10.0)	0.119

* Patients with at least one lesion with FFR ≤ 0.80. TIA—transient ischemic attack, PCI—percutaneous coronary intervention, CABG—coronary artery bypass graft, MI—myocardial infarction, CCS—Canadian Cardiovascular Society. Data are presented as count and proportion (%) or mean ± standard deviation.

**Table 2:** Angiographic characteristics and optical coherence tomography measurements.

Angiographic Characteristics			
	FFR ≤ 0.80 * L = 72	FFR > 0.80 L = 52	
LM	3 (4.17)	2 (3.85)	0.929
LAD	53 (73.61)	21 (40.38)	<0.001
Cx	4 (5.56)	9 (17.3)	0.035
RCA	9 (12.5)	16 (30.77)	0.012
Lesion length (mm)	28.4± 13	17.29 ± 7.5	0.001
Diameter stenosis (%)	53.3 ± 8.7	47.6 ± 8.6	0.036
Proximal reference vessel diameter (mm)	2.78 ± 0.61	3.12 ± 0.57	0.061
Distal reference vessel diameter (mm)	2.3 ± 0.38	2.8 ± 0.6	0.003
A	9 (12.5)	4 (7.7)	0.388
B1	22 (30.56)	21 (40.38)	0.256
B2	19 (26.38)	19 (36.54)	0.226
C	22 (30.56)	8 (15.38)	0.052
Bifurcation	30 (41.67)	21 (40.38)	0.886
Calcification	9 (12.5)	7 (13.46)	0.875
Ostial lesion	2 (2.78)	6 (11.54)	0.05
Severe tortuosity	6 (8.33)	7 (13.46)	0.358
Optical coherence tomography measurements			
Mean lumen area (mm^2^)	3.46 ± 1.29	4.65 ± 2.19	<0.001
MLA (mm^2^)	1.84 ± 0.97	2.66 ± 1.4	<0.001
Mean lesion length (mm)	15.62 ± 9.42	11.8 ± 7.79	0.018
Proximal RLA (mm^2^)	7.27 ± 2.73	9.73 ± 5.31	0.002
Distal RLA (mm^2^)	4.89 ± 1.93	6.85 ± 3.63	<0.001
Calcified plaque	26 (36.11)	19 (36.54)	0.961
Fibrous plaque	24 (33.33)	18 (34.6)	0.882
Lipid-rich plaque	27 (37.5)	15 (28.8)	0.315
TCFA	13 (18.06)	8 (15.38)	0.696
Mean FCT (mm)	0.11 ± 0.07	0.11 ± 0.07	0.882
Minimal FCT (mm)	0.10 ± 0.07	0.10 ± 0.08	0.905
Mean lipid angle (°)	119.59 ± 34.07	104.87 ± 41.03	0.294
Mean angle of the calcium (°)	125.5 ± 68.38	99.54 ± 49.09	0.075
Maximal angle of the calcium (°)	141.73 ± 77.38	113.85 ± 71.59	0.121
Mean cap thickness over the calcium (mm)	0.1 ± 0.07	0.1 ± 0.07	0.882
Calcium volume index * (° × mm)	2780.7 ± 689	1621.6 ± 201	0.306
Lipid volume index ^#^ (° × mm)	1895.7 ± 386	1832.9 ± 283	0.997

LM—left main, RCA—right coronary artery, Cx—circumflex, LAD—left anterior descending, MLA—minimal lumen area, FCT—fibrous cap thickness, RLA—reference lumen area, TCFA—thin cap fibroatheroma. Data are presented as count and proportion (%) or mean ± standard deviation. * calculated as calcium angle multiplied by length specifically for calcified lesions. ^#^ calculated as lipid angle multiplied by length specifically for lipid-rich lesions.

**Table 3:** Correlation between fractional flow reserve and optical coherence tomography measurements.

	Fractional Flow Reserve	
	Pearson Correlation	*p* Value
Mean lumen area (mm^2^)	0.228	0.011
MLA (mm^2^)	0.208	0.02
Mean lesion length (mm)	−0.38	<0.001
Proximal RLA (mm^2^)	0.229	0.017
Distal RLA (mm^2^)	0.292	0.002

MLA—minimal lumen area, RLA—reference lumen area.

## Data Availability

Not applicable.
